# Tumor-Targeting *Salmonella typhimurium* A1-R in Combination with Trastuzumab Eradicates HER-2-Positive Cervical Cancer Cells in Patient-Derived Mouse Models

**DOI:** 10.1371/journal.pone.0120358

**Published:** 2015-06-05

**Authors:** Yukihiko Hiroshima, Yong Zhang, Ming Zhao, Nan Zhang, Takashi Murakami, Ali Maawy, Sumiyuki Mii, Fuminari Uehara, Mako Yamamoto, Shinji Miwa, Shuya Yano, Masashi Momiyama, Ryutaro Mori, Ryusei Matsuyama, Takashi Chishima, Kuniya Tanaka, Yasushi Ichikawa, Michael Bouvet, Itaru Endo, Robert M. Hoffman

**Affiliations:** 1 AntiCancer, Inc., San Diego, California, United States of America; 2 Department of Surgery, University of California San Diego, San Diego, California, United States of America; 3 Department of Gastroenterological Surgery, Yokohama City University Graduate School of Medicine, Yokohama, Japan; Wayne State University, UNITED STATES

## Abstract

We have previously developed mouse models of HER-2-positive cervical cancer. Tumors in nude mice had histological structures similar to the original tumor and were stained by anti-HER-2 antibody in the same pattern as the patient’s cancer. We have also previously developed tumor-targeting *Salmonella typhimurium* A1-R and have demonstrated its efficacy against patient-derived tumor mouse models, both alone and in combination. In the current study, we determined the efficacy of *S*. *typhimurium* A1-R in combination with trastuzumab on a patient-cancer nude-mouse model of HER-2 positive cervical cancer. Mice were randomized to 5 groups and treated as follows: (1) no treatment; (2) carboplatinum (30 mg/kg, ip, weekly, 5 weeks); (3) trastuzumab (20 mg/kg, ip, weekly, 5 weeks); (4) *S*. *typhimurium* A1-R (5 × 10^7^ CFU/body, ip, weekly, 5 weeks); (5) *S*. *typhimurium* A1-R (5 × 10^7^ CFU/body, ip, weekly, 5 weeks) + trastuzumab (20 mg/kg, ip, weekly, 5 weeks). All regimens had significant efficacy compared to the untreated mice. The relative tumor volume of *S*. *typhimurium* A1-R + trastuzumab-treated mice was smaller compared to trastuzumab alone (p = 0.007) and *S*. *typhimurium* A1-R alone (p = 0.039). No significant body weight loss was found compared to the no treatment group except for carboplatinum-treated mice (p = 0.021). Upon histological examination, viable tumor cells were not detected, and replaced by stromal cells in the tumors treated with *S*. *typhimurium* A1-R + trastuzumab. The results of the present study suggest that *S*. *typhimurium* A1-R and trastuzumab in combination are highly effective against HER-2-expressing cervical cancer.

## Introduction

Cervical cancer is the second most common cancer in women [1]. There were 454,000 cases and 200,000 deaths in 2010 worldwide and 11,000 new cases and 3,870 deaths from cervical carcinoma in the U.S. [2, 3]. Paclitaxel, carboplatin, cisplatinum, bleomycin, mitomycin-C, vincristine and irinotecan are used for cervical cancer [4]. However, there is no standard treatment for cervical cancer.

The incidence of HER-2 positivity in cervical cancer was reported from 1% to 21% [5], and overexpression of HER-2 has been associated with more advanced stages and a worse prognosis [6, 7].

We previously developed mouse models of HER-2-positive patient cervical cancer [8]. Our laboratory has also previously developed a genetically-modified bacteria strain, *Salmonella typhimurium* A1-R, selected for tumor-targeting in vivo. *S*. *typhimurium* A1-R is auxotrophic for leu and arg [9]. The strain targets and grows in tumors. In contrast, normal tissue is cleared of these bacteria even in immunodeficient athymic mice.

*S*. *typhimurium* A1-R is effective against prostate cancer [10], breast cancer [11, 12], pancreatic cancer [13–16], glioma [17, 18], lung cancer [19], fibrosarcoma [20, 21], osteosarcoma [22] and ovarian cancer [23].

In the present study, we demonstrate the efficacy of *S*. *typhimurium* A1-R in combination with trastuzumab on mouse models of patient cervical cancer expressing HER-2.

## Materials and Methods

### Ethics Statement

All animal studies were conducted with an AntiCancer Institutional Animal Care and Use Committee (IACUC)-protocol specifically approved for this study and in accordance with the principals and procedures outlined in the National Institute of Health Guide for the Care and Use of Animals under Assurance Number A3873-1. In order to minimize any suffering of the animals the use of anesthesia and analgesics were used for all surgical experiments. Animals were anesthetized by intramuscular injection of a 0.02 ml solution of 20 mg/kg ketamine, 15.2 mg/kg xylazine, and 0.48 mg/kg acepromazine maleate. The response of animals during surgery was monitored to ensure adequate depth of anesthesia. Ibuprofen (7.5 mg/kg orally in drinking water every 24 hours for 7 days post-surgery) was used in order to provide analgesia post-operatively in the surgically-treated animals. The animals were observed on a daily basis and humanely sacrificed by CO_2_ inhalation when they met the following humane endpoint criteria: prostration, skin lesions, significant body weight loss, difficulty breathing, epistaxis, rotational motion and body temperature drop. The use of animals was necessary to understand the in vivo efficacy, in particular, anti-metastatic efficacy of the agents tested. Animals were housed with no more than 5 per cage. Animals were housed in a barrier facility on a high efficiency particulate air (HEPA)-filtered rack under standard conditions of 12-hour light/dark cycles. The animals were fed an autoclaved laboratory rodent diet (Supp. Information S1).

### Animals

Female athymic (*nu/nu*) nude mice (AntiCancer, Inc., San Diego, CA), 4–6 weeks old, were used in this study. Mice were kept in a barrier facility under HEPA filtration. Mice were fed with autoclaved laboratory rodent diet. All mouse surgical procedures and imaging were performed with the animals anesthetized by intramuscular injection of a 0.02 ml solution of 20 mg/kg ketamine, 15.2 mg/kg xylazine, and 0.48 mg/kg acepromazine maleate. All animal studies were conducted with an AntiCancer Institutional Animal Care and Use Committee (IACUC)-protocol specifically approved for this study and in accordance with the principals and procedures outlined in the National Institute of Health Guide for the Care and Use of Animals under Assurance Number A3873-1.

### Specimen collection

The patient provided written informed consent and the tumor specimen was procured under the approval of the Institutional Review Board of the University of California San Diego.

### Subcutaneous implantation of patient cervical cancer

Tumor tissues were obtained from the HER-2-positive cervical cancer patient at surgery and cut into fragments (3-mm^3^) and transplanted subcutaneously in nude mice [8].

### Tissue histology

Tumor tissue was removed along with surrounding normal tissues at the time of resection. The tissues were fixed in 10% formalin and embedded in paraffin before sectioning and staining. Tissue sections (3 μm) were deparaffinized in xylene and rehydrated in an ethanol series. Hematoxylin and eosin (H&E) staining was performed according to standard protocols. For immunohistochemistry, sections (5 μm) were then treated for 30 min with hydrogen peroxide (0.3%) to block endogenous peroxidase activity. The sections were subsequently washed with PBS and unmasked in citrate antigen-unmasking solution (Mitsubishi Kagaku Iatron, Inc., Tokyo, Japan) in a water bath for 40 min at 98°C. After incubation with 10% normal goat serum, the sections were incubated with anti-HER-2/ErbB2 (1:100; Cell Signaling Technology, Inc., Danvers, MA, USA) at 4°C overnight. The binding of primary antibodies was detected using anti-rabbit secondary antibodies and an avidin/biotin/horseradish peroxidase complex (DAKO Cytomation, Kyoto, Japan) for 30 min at room temperature. The labeled antigens were visualized with the DAB kit (DAKO Cytomation). Finally, the sections were counterstained with hematoxylin and examined with a BH-2 microscope (Olympus, Tokyo, Japan) equipped with an INFINITY1 2.0 megapixel CMOS digital camera (Lumenera Corporation, Ottawa, Canada). All images were acquired using INFINITY ANALYZE software (Lumenera Corporation) without post-acquisition processing.

### Treatment of patient cervical cancer growing in nude mice

Six weeks after implantation, the mice were randomized to 5 groups and treated as follows: (1) no treatment; (2) carboplatinum (Selleck Chemicals, Houston, TX, USA, 30 mg/kg, ip, weekly, 5 weeks); (3) trastuzumab (Genentech, Inc., South San Francisco, CA, USA, 20 mg/kg, ip, weekly, 5 weeks); (4) *S*. *typhimurium* A1-R (5 × 10^7^ CFU/body, ip, weekly, 5 weeks); and (5) *S*. *typhimurium* A1-R (5 × 10^7^ CFU/body, ip, weekly, 5 weeks) + trastuzumab (20 mg/kg, ip, weekly, 5 weeks) were co-administered. Each treatment arm comprised 6 tumor-bearing mice. Tumor size was evaluated every 3 or 4 days by caliper measurements and the approximate volume of the tumor was calculated using the formula 4/3π (d/2)^2^ D/2; where d is the minor tumor axis and D is the major tumor axis. Body weight of the mice was measured on a balance every 3 or 4 days. Relative tumor volume and body weight were calculated by comparison to day-1 values. Tumors were imaged with a Canon EOS 60D digital camera with an EF–S18–55 IS lens (Canon, Tokyo, Japan) and harvested for analysis.

### Evaluation of histopathological response

Histopathological response to chemotherapy was defined according to Evans’s grading scheme: Grade I, little (<10%) or no tumor cell destruction is evident; Grade II a, destruction of 10%-50% of tumor cells; Grade II b, destruction of 51%-90% of tumor cells; Grade III, few (<10%) viable-appearing tumor cells are present; Grade IV, no viable tumor cells are present [24, 25].

### Statistical analysis

PASW Statistics 18.0 (SPSS, Inc) was used for all statistical analyses. Final tumor volumes (at day-36) in each treatment group were compared to the untreated control using a 2-tailed Student’s *t*-test. A p-value of ≤ 0.05 was considered statistically significant for all comparisons.

## Results and Discussion

### Histology of the original tumor is preserved in the mouse

**Fig 1:**
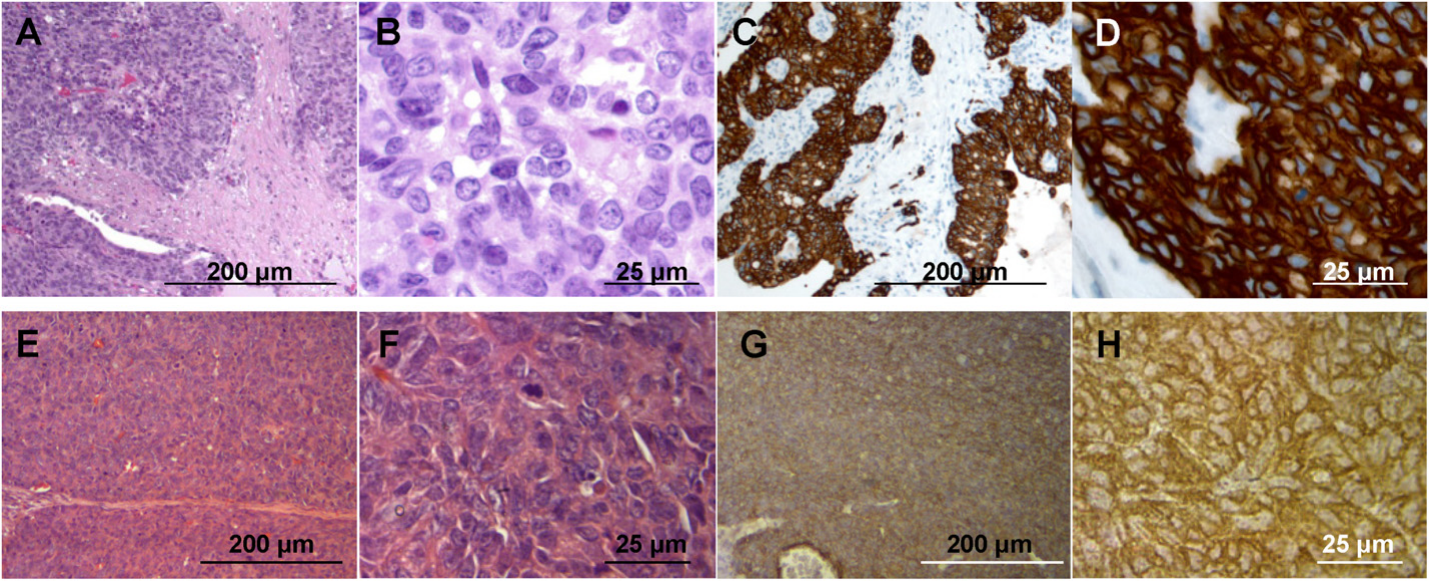
Tumor histology and immunohistochemistry. (A) H & E-stained section of the original patient tumor. (B) High magnification image of (A). (C) Immunostained section of the original patient tumor using anti-HER-2 antibody. (D) High magnification image of (C). (E) H & E-stained section of the mouse-grown tumor. (B) High magnification image of (F). (G) Immunostained section of the mouse-grown tumor using an anti-HER-2 antibody. (H) High magnification image of (G). Scale bars: 200 μm (A, C, E and G) and 25 μm (B, D, F and H).

Sheet-like growth without gland formation and stromal tissue with fibroblastic proliferation, which penetrated into nests of cancer cells was observed in the H&E stained sections of the original tumor (Fig 1A). Oval- to spindle-shaped cancer cells with high nuclear/cytoplasmic ratio were found in high magnification images (Fig 1B). In the immunostained sections with anti-HER-2 antibody, the membrane and the cytoplasm of cancer cells were strongly stained, but no staining was found in the stromal tissue (Fig 1C and 1D). All mouse-grown cervical patient-derived tumor had histological structures similar to the original tumor and were stained by anti-human HER-2 antibody (Fig 1E–1H), suggesting that the model recapitulates the biological behavior of the original tumor [8].

### *S*. *typhimurium* A1-R + trastuzumab combination is effective against patient-derived cervical cancer growing in nude mice

**Fig 2:**
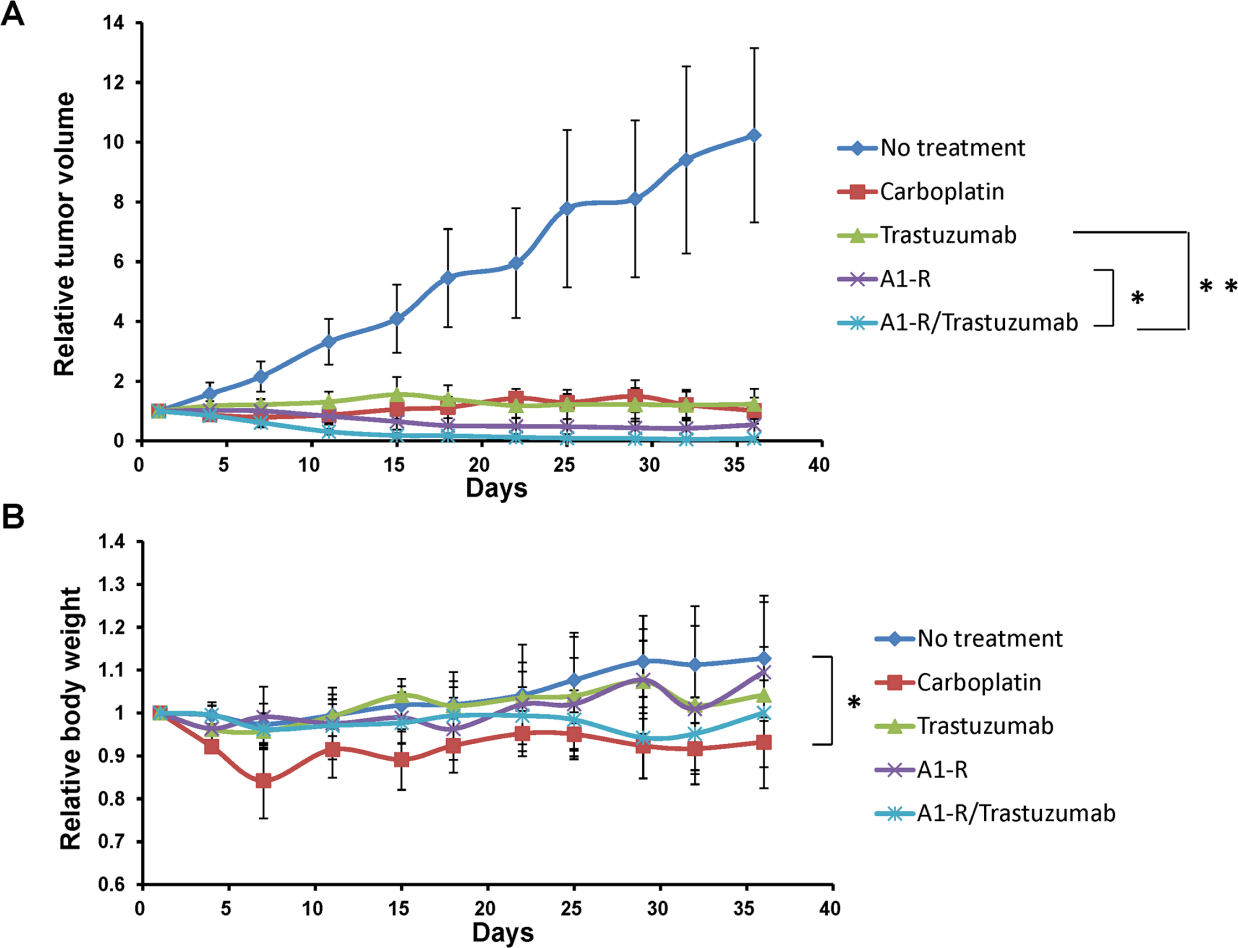
*S*. *typhimurium* A1-R, trastuzumab and combination drug treatment of the patient cervical tumor grown in nude mice. (A) Growth curves of the subcutaneous tumor treated with various drugs. The values are mean relative tumor volumes ± S.D. (bars) of five different tumors. * p < 0.05, ** p < 0.01. (B) Body weight curves of the mice with the subcutaneous tumors treated with the indicated drugs. The values are mean relative body weights ± S.D. (bars) of five different mice.

The relative tumor volumes at day-36, compared to day-1, of each group were as follows: (1) no treatment: 10.23 ± 2.91; (2) carboplatinum: 1.02 ± 0.43; (3) trastuzumab: 1.23 ± 0.50; (4) *S*. *typhimurium* A1-R: 0.54 ± 0.40; (5) *S*. *typhimurium* A1-R + trastuzumab: 0.08 ± 0.04 (Fig 2A). All regimens had significant efficacy compared to the untreated mice. The relative tumor volume of *S*. *typhimurium* A1-R + trastuzumab-treated mice was smaller than with trastuzumab treatment alone (p = 0.007) or *S*. *typhimurium* A1-R treatment alone (p = 0.039) (Fig 2A).

The relative body weight at day-36, compared to day-1, of each group was as follows: (1) no treatment: 1.13 ± 0.14; (2) carboplatinum: 0.93 ± 0.06; (3) trastuzumab: 1.04 ± 0.22; (4) *S*. *typhimurium* A1-R: 1.09 ± 0.06; (5) *S*. *typhimurium* A1-R + trastuzumab: 1.00 ± 0.08. No significant body weight loss was found compared to the untreated mice, except for carboplatinum-treated mice (p = 0.021) (Fig 2B).

### *S*. *typhimurium* A1-R + trastuzumab combination eradicates HER-2-positive cervical cancer cells in mice as observed in histological sections

**Fig 3:**
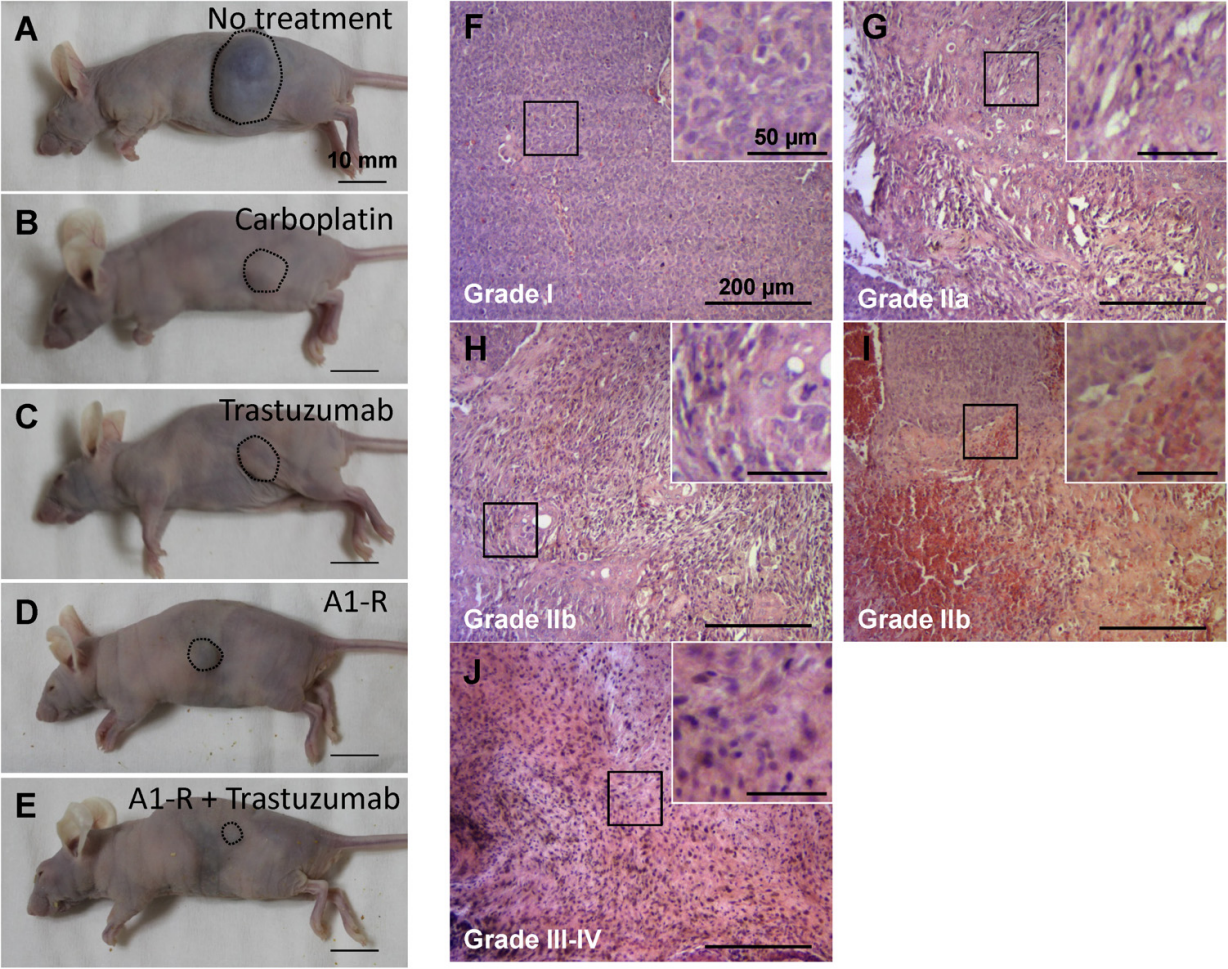
Efficacy of *S*. *typhimurium* A1-R, trastuzumab and combination treatment on tumor size and histology. (A) Untreated control. (B) Carboplatinum-treated mice. (C) Trastuzumab-treated mice. (D) *S*. *typhimurium* A1-R-treated mice. (E) *S*. *typhimurium* A1-R + trastuzumab-treated mice. These images were obtained at day-36. Scale bars: 10 mm. Histopathological response to treatment was defined according to Evans’s grading scheme. Treatment effect of untreated control (F) was judged as grade I; carboplatinum (G) as IIa; trastuzumab (H) as IIb; *S*. *typhimurium* A1-R (I) as IIb; and *S*. *typhimurium* A1-R + trastuzumab (J) as III—IV. Scale bars: 200 μm.

Histopathological response to treatment was defined according to Evans’s grading scheme. In tissue sections from untreated mice, numerous cancer cells were observed (Fig 3F). Approximately 30% of the cancer cells were destroyed and replaced by stromal cells in tissues sections from mice treated with carboplatinum (Fig 3G). Seventy percent of the cancer cells were destroyed in the tissue sections from mice treated with trastuzumab (Fig 3H). Sixty percent of the cancer cells were destroyed in the tissue sections from mice treated with *S*. *typhimurium* A1-R (Fig 3I). In the tissue sections from mice treated with *S*. *typhimurium* A1-R + trastuzumab, viable cancer cells were not detected and were replaced by stromal cells (Fig 3J). The untreated control was judged as grade I; carboplatinum treatment as IIa; trastuzumab treatment as IIb; *S*. *typhimurium* A1-R treatment as IIb; and *S*. *typhimurium* A1-R + trastuzumab treatment as III—IV. These results suggest the *S*. *typhimurium* A1-R + trastuzumab combination can eradicate HER-2-positive cervical cancer in mice.

The mechanisms of the strong efficacy of the combination of *S*. *typhimurium* A1-R and trastuzumab remains to be elucidated. One contributing factor could be the ability of *S*. *typhimurium* A1-R to stimulate (decoy) the cell cycle of quiescent, resistant cells, which then begin to cycle and become sensitive to trastuzumab [26]. Having found the strong efficacy of the combination of *S*. *typhimurium* A1-R and trastuzumab in subcutaneous models, future experiments will utilize orthotopic models, using the surgical orthotopic implantation (SOI) method our laboratory has developed for patient tumors [27–32]. Access of *S*. *typhimurium* A1-R to orthotopic tumors has been previously demonstrated by us [8–24]. Access to trastuzumab therapy in orthotopic models has already been demonstrated by us as well [33]. The orthothopic models of a series of HER-2 positive and mixed HER-2 expressing cervical cancer will allow efficacy testing of the combination of *S*. *typhimurium* A1-R and trastuzumab against primary and metastatic disease and further characterization of stromal and cancer cells before and after treatment, as well as the effects of leukocyte-depleting regimens following or before the treatment. SOI requires surgery and since ketamine may affect the immune system, different anesthetics will be compared for any effect on treatment response in future experiments.
